# Management of Asymptomatic Patients With Textured Surface Breast Implants

**DOI:** 10.1093/asjof/ojz025

**Published:** 2019-08-22

**Authors:** Patricia A McGuire, Anand K Deva, Caroline A Glicksman, William P Adams, Melinda J Haws

**Affiliations:** 1 Department of Plastic and Reconstructive Surgery, Macquarie University, Sydney, Australia; 2 Clinical Assistant Professor of Plastic Surgery, Hackensack Meridian School of Medicine at Seton Hall, Nutley, NJ; 3 Department of Plastic Surgery, and Program Director of the Aesthetic Surgery Fellowship at UTSW, University of Texas Southwestern, Dallas, TX; 4 Clinical Assistant Professor of Plastic Surgery, Vanderbilt University, Nashville, TN

## Abstract

With the recent voluntary recall by Allergan of their Biocell textured implants, many plastic surgeons are left with questions of how to best manage asymptomatic patients who have concerns about having these devices. We realized that there is no clear, published recommendations or scientific data to guide surgeons on how to discuss options with their patients and recommendations for surgical management in this uncharted territory. Using available literature and personal experience, we answer the most common questions we are hearing from our plastic surgery colleagues.

The voluntary recall by Allergan of their Biocell textured breast implants has left surgeons with questions about the best way to manage these patients.^1^ Breast implant-associated anaplastic large cell lymphoma (BIA-ALCL) remains rare^2^ and there are no definitive scientific studies regarding management of asymptomatic patients with textured implants, and no current method to determine which patients who have textured implants of any brand may be at higher risk. The most important thing to do for these patients is to educate them about BIA-ALCL, so they understand the actual risk, recognize signs and symptoms, and seek medical evaluation for the best chance of early diagnosis and cure in the rare instance that they develop ALCL.^3^ Plastic surgeons must be comfortable with the assessment of textured breast implant patients and up to date in their knowledge of the presentation, work-up, National Comprehensive Cancer Network (NCCN) guidelines for evaluation/staging, diagnosis, and treatment of BIA-ALCL.^3^

1. How do you manage an asymptomatic patient with a textured implant who is concerned about developing ALCL?
  - Patients need to be evaluated for a history of any change to her breast, specifically those related to ALCL, swelling, mass, pain, or rash. Any symptoms need to be evaluated with physical examination, ultrasound, mammogram, or MRI as indicated.^2^
  - If the physical examination and testing are negative, the patient should be reassured that nothing has been found physically or radiologically to indicate BIA-ALCL. These patients should be offered regular follow-up visits and educated on the signs/symptoms of ALCL and instructed to return sooner for any change in their breast.
2. Should asymptomatic patients have their implants removed prophylactically?
  - None of the regulators worldwide are recommending prophylactic removal. The FDA has specifically stated “If you have no symptoms, we are not recommending the removal of these or other types of breast implants due to the low risk of developing BIA-ALCL.” ^1^
  - For patients who are concerned, evaluate the patient for signs/symptoms of ALCL and test appropriately as indicated.^2^
  - If the patient is asymptomatic, offer to see the patient for regular follow-up with exam and imaging as indicated.
3. If an asymptomatic patient does elect to have their implants removed/replaced is a total or partial capsulectomy required?
  - There is no scientific data to support complete removal of an implant capsule in the absence of malignancy or capsular contracture.^4^
  - If a capsulectomy is performed, both solid tissue and any seroma fluid (if present) should be sent for pathological examination whenever exchange or removal is performed for any indication.^3^
  - Capsulectomy carries risk(s) and informed, educated consent should be obtained after discussing the potential risks with the patient.^4^
  - For patients who elect to undergo textured implant removal, with or without replacement, because of anxiety related to their textured implant and potential for development of ALCL, the aim is to perform a precise complete or partial capsulectomy unless intraoperative findings do not allow this to be performed safely.^5^
  - The capsule should be photographed to document its appearance, and the capsule sent to pathology for examination with cultures performed if indicated or requested by the patient.
  - Any patient with symptoms, swelling, seroma, mass, rash, etc., should have appropriate preoperative work-up with aspiration of fluid, cytology, immunohistochemistry testing for CD30, and cultures as indicated per NCCN guidelines for diagnosis of BIA-ALCL.^3^
4. Should a capsulectomy be performed at the time of exchange from textured expander to smooth implant?
  - In the absence of seroma, mass in the capsule, or any other abnormality encountered, removal of the capsule is not necessary unless clinically indicated.
5. Has there been a case of ALCL in a patient who had an uncomplicated textured expander to smooth implant?
  - Anecdotally, we are aware of a case, but this is yet to be confirmed and/or published.
6. Should acellular dermal matrix (ADM)/mesh be removed if a capsulectomy is being performed in an asymptomatic patient?
  - ADM or mesh that was placed at previous surgery should only be removed if it is not incorporated or as part of an en bloc capsulectomy where tissue and implant are removed together in to obtain clear margins for ALCL or other malignancy as indicated.^3^
7. If capsulectomy is performed should the capsule be sent to pathology? What testing should be performed? Is CD30 testing necessary?
  - Any capsule which is removed, especially if it is in any way abnormal, should be sent to pathology for microscopic evaluation with further testing as indicated. Expert pathologists do not believe that in the absence of suspicious morphology, CD30 stain is needed for examination of a capsule. (personal communication, Dr. Marshall Kadin, April 24, 2019). There has been a social spread of misinformation recommending sending the solid capsule for CD30.^6^ The solid tissue pathologic evaluation is the only test needed after capsulectomy unless abnormalities are found, then further testing will be considered per the pathologist’s recommendations.^3^ CD30 is a normal cell marker, not specifically related to BIA-ALCL. There can be CD30 positive lymphocytes in a normal lymphocyte population. CD30 markers are significant when found in abnormal lymphocytes, and specifically with a single clone of CD30+ ALK-anaplastic lymphocytes.^7^ We recommend for any concerns, consultation with the pathologist be considered prior to surgery.
  - Capsule tissue should be sent in formalin unless the pathologist will be performing cultures of the tissue or if the pathologist requests a fresh specimen after preoperative consultation.
  - Any seroma fluid should be sent for cytology and immunohistochemistry for CD30 testing.
  - For implants placed for cosmetic reasons, insurance may not cover exam by a pathologist, so informed financial consent for testing should be obtained preoperatively.
8. Has there been a case of an ALCL in a patient who had implant removal without capsulectomy?
  - There have been cases where ALCL was diagnosed after implant removal without capsulectomy. All cases had a history of seroma at the time of implant removal, which had not been tested for ALCL, it is felt that these cases were undiagnosed ALCL at the time of implant removal.^8^
  - All ALCL cases that have been reported where a smooth implant was in place at the time of diagnosis, had a previous history of a textured implant, mixed implant history, or unknown implant history. The details of those cases are unclear as to whether a capsulectomy was or was not performed. For that reason, removal of the capsule should be considered, if it can be safely performed to potentially lower the risk of developing BIA-ALCL.^9^
9. Allergan announced a voluntary recall of Biocell implants on July 24, 2019. What does that mean? Why would they use a word that could cause such anxiety for our patients?
  - Recall is a regulatory term used for when a manufacturer takes a correction or removal action to address a problem with a medical device that violates FDA regulations. Recalls occur when a medical device is defective, when it could be a risk to health, or when it is both defective and a risk to health.^10^
  - This recall involved a voluntary suspension of sales, shipments, and removal of devices from operating rooms and physician offices.
  - This does not mean Biocell implants that are already implanted should be removed.

BIA-ALCL is an ever-changing disease, and it is difficult to give patients an accurate risk because the numbers continue to change. These recommendations are also subject to change as more data are collected and ALCL is further studied.^1^
